# Supplementary material for: Are carbon emissions trading and green financial instruments synergistic? -Comprehensive quantitative research based on content analysis
Source: PLoS One. 2024 Mar 7;19(3):e0298601. doi: 10.1371/journal.pone.0298601 (PMC10919601; doi:10.1371/journal.pone.0298601)
Supplement: S1 Data — (ZIP) [file pone.0298601.s001.zip › Data/地级市Z_F工作报告.zip!/║╙▒▒/╨╧╠¿╩╨/└┤╫╘╣1⁄2╓┌║┼í╛┬φ┐╦╩2╛▌═°í┐_2007.docx]

各位代表：

　　现在，我代表邢台市人民政府向大会作工作报告，请予审议，并请市政协委员和列席会议的同志提出意见。

　　一、2006年工作回顾

　　2006年，是“十一五”开局之年。全市人民在省委、省政府和市委的领导下，高举邓小平理论和“三个代表”重要思想伟大旗帜，全面落实科学发展观，坚持 “全党抓经济、突出抓项目、强力抓环境”的要求，团结奋斗，开拓进取，各项工作实现了新突破，圆满完成了市十二届人大四次会议确定的任务。初步核算，全市生产总值完成790亿元，同比增长13%，其中一、二、三产分别增长5.2%、15.8%和12.3%;全部财政收入突破70亿元，可比增长18.2%，其中地方一般预算收入25.5亿元，可比增长19.8%;城镇居民人均可支配收入9020元，农民人均纯收入3542元，分别增长16.4%和8%，实现了起好步、开好局的目标。

　　是大力发展县域经济，综合实力不断壮大。把发展县域经济作为全市经济的重要支撑来抓，从各地发展阶段和实际情况出发，实施分类指导。各县市立足自身优势，围绕项目建设、开放招商、培育主导产业、园区建设和创建文明生态村等，增强机遇意识，创新工作思路，强化工作措施，县域经济的发展速度加快，发展质量提高，支撑能力增强。县县有了投资5000万元以上的项目，多数县有了投资亿元以上的项目；一批传统产业得到明显提升，产业集中度进一步提高；民营经济不断壮大，保持了快速健康的发展势头；城镇建设力度加大，辐射带动作用进一步增强；与新农村建设相结合，文明生态村创建活动深入开展，25%的村基本建成文明生态村。

　　二是调整优化经济结构，主导产业支撑能力提高。以农民增收为核心，加大了农业结构调整力度，畜牧、蔬菜、林果三大主导产业占大农业的比重达56%，其中畜牧业产值占农业总产值的比重达到41%；农业产业化经营取得新进展，新增年销售收入5000万元以上的龙头企业4家，农业产业化经营率提高0.9个百分点；全年粮食总产374万吨，同比增长2.9%，肉蛋奶等农副产品产量进一步增加。以解决“有企业无产业、有产业无企业”为切入点，加快了工业结构调整步伐，围绕骨干企业，延长产业链条，发展优势产业，产品科技含量和附加值不断提高，涌现出一批名牌产品，核心竞争力逐步增强。以邢钢线材制品加工基地为龙头的钢制品产业，建设了宁波工业园、振成机械等一批项目，产业规模不断扩张；以建滔、旭阳为龙头的煤化工产业，开工建设了深加工项目，产业链条不断延伸，促进了产品加工增值；以宁晋晶龙为龙头的硅材料产业，企业规模扩大，产品档次提高，形成了从单晶硅棒材到生产太阳能电池片较为完整的产业链；机械制造、纺织服装、食品、医药、建材等产业也都实现了新的发展。围绕传统特色产业，引进和培育了一批带动作用明显的骨干龙头企业，促进了产业升级。如平乡自行车与印尼、台湾客商合资建设了整车生产项目；临西新上了技术含量高的高速轴承，提升了产业水平和竞争力。全市规模以上工业完成增加值241亿元，同比增长 19.8%，实现利税102亿元，增长30.2%。以优化生产力布局结构为方向，规划启动了“一带两翼”发展战略，从临城到沙河的百里工业经济带，初步完成了发展规划，正在谋划建设一批工业园区、物流园区和生态园区；围绕打造西部百里生态太行和发展壮大东部黑龙港流域特色产业，谋划建设了一批项目，各项工作顺利推进。以发展外向型经济为导向，积极扩大对外贸易，全年完成进出口总值8.6亿美元，增长18％，其中出口6.5亿美元，增长20.7％。以改造提升传统服务业、发展现代服务业和旅游业为重点，加快发展第三产业，全年完成社会消费品零售总额231.7亿元，增长15%。

　　三是招商引资成效显著，项目建设势头强劲。坚持把项目建设作为带动全市经济社会发展的龙头，把招商引资作为加快发展的突破口，开展了项目建设年和开放招商活动，制定出台了一系列优惠政策和激励约束措施，全年有两个月定为无会月，各级集中精力开展招商引资活动，县市领导带头招商，全市上下抓招商、上项目、求发展的积极性空前高涨。各部门发挥职能作用，主动服务大局，解决了资金、土地等大量难题，全年争取省批建设用地1.5万亩；各类金融机构新增贷款比年初增长11%。全市招商引资和项目建设取得了明显成效，全年实际利用外资1.6亿美元，同比增长15.4％，引进市外国内资金59亿元，增长20.4％。全社会固定资产投资完成406亿元，同比增长35%；全市230个重点建设项目，全年完成投资230亿元，增长10%。新开工项目增多，已开工项目143个，同比提高20%；竣工项目增多，德龙技改、宁晋太阳能电池、威县生物发电、南宫千喜鹤等54个项目竣工投产，比上年增加21个；新谋划项目增多，谋划了投资亿元以上项目150个，总投资1487亿元，其中投资30亿元以上的项目14个，总投资860亿元。

　　四是各项改革不断深化，发展活力进一步增强。把国企改革与招商引资结合起来，积极吸引外来要素和民营资本参与国企改革。全市23家大中型企业，已有21家完成或进入改制程序，邢机由中钢集团并购，鲸鱼轮胎由美国GPX公司收购。粮食购销企业改革顺利推进，通过兼并、重组，企业数量减少了163家，清理挂账 5.7亿元。不断深化财政预算管理、县乡财政体制等项改革，稳步推进行政管理体制改革，严格控制财政供养人员数量，公共管理和社会服务职能得到强化。市直商贸、物资、供销企业改制和农村综合改革等稳步推进。引入竞争机制，实行多元化投资，积极探索了教育、卫生、文化等社会事业领域的改革。

　　五是切实加强城镇建设，城市化水平有了新提高。研究出台了城市规划、建设、管理等方面的一系列政策规定，保证了城建工作的顺利开展。中心城市在完成总体规划修编的基础上，先后完成或基本完成了综合交通、绿地系统、古城保护等5类24项规划的修编，规划滞后的局面得到改变，城市框架进一步拉大。实施了一批重点工程，七里河综合治理全面启动，投入资金5亿多元，基本完成了河道清障，共清理土地1万亩，清除各类建筑物近10万平方米，钢铁路大桥建成通车；扩建改造主要街道16条；公园和街心游园拆除围栏向群众开放；投资1亿多元，完成围寨河治理退污还清工程；新增集中供热面积200万平方米，发展居民燃气用户1 万户。城市管理水平有所提高，市容市貌有了新变化，新增绿地面积180万平方米，人均公共绿地面积达到6.6平方米，绿化覆盖率达到35.5%。围绕创建省级环保模范城，加大了环境治理力度，关闭整合市区周边“四小”污染企业取得阶段性成效，拆除集中供热区内燃煤锅炉81台，市区空气质量二级及好于二级天数达到281天，比上年增加16天。小城镇建设有9个县城镇规划得到批复，规划面积扩大了近一倍，拓展了城镇发展空间；围绕提高要素承载能力，改善居民生活质量，注重完善城镇功能，开工建设了路网、供热供气、医院、学校、公园等基础设施项目；全市城市化率达到36%，提高1.2个百分点。交通体系进一步完善，完成投资17亿元，建设了邢临、青银高速公路连接线，完成国省干线公路改造193公里；新建县乡村公路1255公里，新增通油路村181个，全市通油路村达到96%。

　　六是努力构建和谐邢台，社会事业全面发展。从解决人民群众最现实、最关心、最直接的问题入手，大力实施“民心工程”，促进了各项社会事业的发展。积极扩大就业再就业，全市城镇新增就业3.8万人，城镇登记失业率低于省控目标，转移农村富余劳动力15.3万人次。加强社会保障工作，养老、失业、医疗、工伤保险覆盖面进一步扩大，城镇低保实现了动态管理下的应保尽保，共发放保障金5485万元，年底保障人数7.2万人；农村低保工作全面铺开，共发放低保金 1321万元；实施了城乡医疗救助制度，共救助城镇和农村困难群众13.7万人。重视扶贫和救灾救济工作，争取上级扶贫资金4137万元，解决了5万人口的温饱问题；争取救灾资金1200万元，救助灾民13.7万人，解决了4.8万人的饮水安全问题。加快科技进步，安排科技资金452万元，实施科技项目 69项，完成国内先进水平以上科技成果60项。大力发展教育事业，优化农村中小学布局，撤并中小学212所，改造中小学95所，新建校舍13万平方米；认真落实“两免一补”政策，落实资金3300多万元，近18万学生得到资助；“普九”教育继续巩固提高，高中段毛入学率达到60%；职业教育和成人教育成绩显著，邢台技师学院挂牌成立，向非农产业和沿海地区转移劳动力8.9万人；高等教育不断进步，邢台学院获得学士学位授予权，形成了“一本四专”的高教格局。加大计划生育集中整治力度，完成了省下达的人口控制目标，计划生育工作跨入省先进行列。卫生事业加快发展，投资2748万元，改扩建36个乡镇卫生院和4个县级医院；完成了16个县级疾控中心和9个传染病病区项目；宁晋、邢台县新型合作医疗试点工作扎实推进，参合人数70多万，补偿费用1800万元。精神文明创建活动深入开展，举办了邢台抗震40周年纪念活动，新建了董振堂纪念馆，爱国主义精神和社会主义道德得到进一步弘扬；切实加强国防动员工作， “双拥”共建取得新的成果。繁荣文化体育事业，继续开展了申报国家级历史文化名城准备工作，启动了大开元寺修复工程；建成了27个文化信息资源中心，群众精神文化生活日益丰富；全民体育健身活动蓬勃开展，竞技体育水平不断提高。高度重视社会稳定工作，加大了安全生产工作力度，事故发生起数和死亡人数实现了双下降，全市地方煤矿、非煤矿山、危险化学品、烟花爆竹等重点行业没有发生重大以上事故。在全市192个乡镇建设了信访服务中心，建立完善了“三位一体” 矛盾纠纷调解机制，妥善解决了人民内部矛盾；强化社会治安综合治理，加强了市区治安防控体系建设，严厉打击各种刑事犯罪，保持了社会稳定。民族宗教、人事、审计、统计、广播电视、残疾人、人防、气象、档案、地方志等工作也都取得了新成绩。

　　七是围绕加强决策力、执行力、控制力，增强了落实科学发展观的自觉性。正确把握宏观经济形势的变化，强化了对重大战略性问题的研究，对科学发展观的理解更加深刻，增强了贯彻落实的自觉性。中央出台了新的宏观调控政策后，从我市实际出发，把落实科学发展观作为承接产业转移、实现跨越式发展的机遇，既坚持了积极主动、有所作为，加大项目建设和招商引资力度，加快发展步伐；又以对历史、对人民负责的态度，注重了经济社会的可持续发展，严把环保准入关，防止“饥不择食”，提高了项目建设质量，促进了经济结构调整和增长方式转变。加强政府法制建设，建立了全市突发事件应急机制，有效履行了政府职能。优化发展环境，强化机关效能建设，深入开展了民主评议行风活动，行政提速、服务提质取得了新成效。狠抓市行政服务中心的规范运行和县级行政服务中心筹建启动，提高了办事效率和服务质量。自觉接受人大的法律监督、工作监督和政协的民主监督，全年共办理人大代表建议202件、政协提案503件。加强廉政建设，认真落实廉洁自律的各项规定，查处了一批违法违纪案件。以抓落实为核心，切实转变工作作风，出台实施问责制八条措施，推动了各项工作的顺利开展。

　　各位代表，一年来我们取得的成绩，是省委、省政府和中共邢台市委正确领导的结果，是人大、政协依法监督和大力支持的结果，是全市人民共同努力的结果。在此，我代表市人民政府，向全市广大工人、农民、知识分子、各级干部和社会各阶层的建设者，向人大代表、政协委员，向各民主党派、工商联、无党派人士和人民团体，向驻邢人民解放军、武警官兵和政法干警，向中省直机关驻邢各单位，向关心和支持我市发展的国内外朋友，致以崇高的敬意和衷心的感谢！

　　在看到成绩的同时，我们也清醒地认识到，我市经济建设和社会生活中还存在不少问题和薄弱环节。产业结构优化度低，部分企业竞争力弱，应对市场风险能力差；大项目、好项目偏少，高科技项目更少；农民增收难度加大，增收形势不容乐观；就业再;业和社会保障压力加大，“两个确保”基础比较脆弱；城市化水平还比较低；社会事业投入不足，历史“欠帐”较多；政府工作方式和作风与新形势新任务的要求还不适应，发展环境有待进一步优化，特别是个别机关还存在着形式主义、官僚主义的现象，一些公务人员不作为、乱作为和以权谋私的问题时有发生等等。对这些问题，我们将高度重视，在今后工作中采取有力措施认真加以解决。

　　二、2007年发展思路和目标

　　当前，总体形势有利于我们加快发展。科学发展、和谐发展深入人心，国际国内产业转移和技术扩散加快，“环渤海”加速崛起，建设京津冀都市圈，给我们带来了难得的发展机遇。党的十六大以来，全市经济实力进一步壮大，干部群众加快发展、干事创业的热情高涨。特别是党的“十七大”将要召开，建设沿海强省开始起步，市委七届二次全会作出了《在建设沿海经济社会发展强省中加快实现富民强县兴市目标的决定》，在全市上下统一了思想，形成了共识，凝聚了力量，为加快发展奠定了坚实的思想基础。只要我们进一步解放思想，开拓进取，牢固树立沿海意识、开放意识、机遇意识，坚持正确的发展思路不动摇、强有力的工作措施不松懈、加快发展的机遇不错过，通过三至五年的努力就能够实现超常规跨越式发展。

　　今年政府工作的总体要求是：以邓小平理论和“三个代表”重要思想为指导，认真贯彻省、市第七次党代会精神，高举科学发展大旗，持之以恒地弘扬“树正气、讲团结、求发展”主旋律，持之以恒地贯彻“全党抓经济、突出抓项目、强力抓环境”方针，围绕实现更好更快发展、构建和谐邢台两大任务，树立沿海新意识，抢抓发展新机遇，打造邢台新优势，实施工业突破、大项目突破、开放招商突破、产业园区突破、城市化突破、改革创新突破战略，更加注重调整经济结构和转变增长方式，更加注重资源节约和环境保护，更加注重改革开放和自主创新，更加注重统筹城乡和区域协调发展，更加注重促进社会发展和解决民生问题，在建设沿海经济社会发展强省中加快实现“富民、强县、兴市”目标。

　　按照上述要求，今年全市经济社会发展的主要预期目标是：生产总值增长13%以上；单位生产总值能耗下降4%以上；主要污染物排放总量减少3%以上；全部财政收入和地方一般预算收入均增长16%以上；全社会固定资产投资增长25%以上；规模以上工业增加值增长20%以上；实际利用外资和引进国内市外资金均增长15%以上；进出口总值增长15%；社会消费品零售总额增长15%；城镇居民人均可支配收入增长11%，农民人均纯收入增长7%。做好今年工作，我们应把握好以下几个方面：

　　第一，坚持把实现更好更快发展作为主题。邢台作为欠发达地区，面临着补课和赶超的双重压力，加快发展是第一位的任务，实现“快”，“好”是先决条件，没有 “好”的“快”，是低效、低质的“快”，是不可持续的“快”，就容易走弯路，甚至付出巨大的代价，“快”也就难以为继。工作中，我们要把更好更快发展作为一个有机统一的整体，好中求快，快中见好，切实把经济社会发展转入科学发展的轨道。

　　第二，坚持积极、有作为地贯彻落实中央宏观调控政策。中央加强和改善宏观调控的目的，是保持经济持续快速健康发展。我们必须从邢台实际出发，坚持实事求是、分类指导、区别对待、有保有压的原则，坚决遏制盲目投资和不合理重复建设，切忌那种“有项目就行，能发展就好”的粗放式发展思路和模式，保持合理投资规模，在加大投入中优化投向，在加快发展中转变增长方式，在调优增量中调强存量，在加快发展速度中提高发展质量。

　　第三，坚持把调整经济结构和转变增长方式作为紧迫任务。提高全市经济综合竞争力，必须调整优化结构、转变增长方式。依托骨干企业，延长产业链条，形成优势产业；引进培育龙头骨干企业，带动提升传统产业；在承接产业转移中优化产业结构、产品结构、企业组织结构和资本结构，提高产业集中度，增强企业竞争力。把节能降耗、保护环境、集约用地、资源整合、发展循环经济作为转变经济增长方式的突破口和重要抓手，推进资源节约型和环境友好型社会建设。

　　第四，坚持把改革开放和科技创新作为根本动力。我市要实现跨越式发展，活力在改革，出路在开放，支撑在科技。我们必须破除各种思想和体制障碍，为更好更快发展提供体制机制保障；强化机遇意识，以更宽的视野、更大的气魄，扩大开放招商，承接产业转移；积极采用高新技术和先进适用技术提升产业企业发展水平，增强经济发展的技术支撑力。

　　第五，坚持把统筹城乡协调发展放在活跃全局的位置。以项目建设、产业集聚、园区建设为载体，深入推进工业化、城市化、农业产业化“三化”互动，加快建立以工促农、以城带乡的长效机制，把挖掘农业自身潜力与工业反哺农业结合起来，把扩大农村就业与农村劳动力向城镇转移结合起来，把推进社会主义新农村建设与加快城市化进程结合起来，实现城乡协调发展、共同进步。

　　第六，坚持把解决民生问题作为构建和谐邢台的着力点。社会和谐，民生是基础。从满足人民群众日益增长的物质文化需求出发，谋发展、促发展；从人民群众最要需解决的问题入手，办实事、解民忧，努力形成全市人民各尽其能、各得其所而又和谐相处的局面。

　　三、促进经济更好更快发展

　　加快实现“富民、强县、兴市”目标，首要问题是增强综合经济实力。我们必须始终坚持以经济建设为中心，突出重点，狠抓关键，带动全局，努力实现经济持续快速健康发展。

　　（一）实施工业突破战略，推进经济加快发展。邢台欠发达的原因在于工业发展慢，我们要实现追赶型、跨越式、超常规发展，潜力、希望和出路在工业。无论是提高城乡人民收入、增加就业岗位，还是发展社会事业、加快城市化进程、建设和谐社会等，都离不开工业经济的支撑。因此，我们要把经济工作的主要精力用到实施工业突破上，把实施工业突破战略作为振兴全市经济的“牛鼻子”和突破口。从今年到2010年，年年都是工业突破年，年年都是项目建设年，年年都是开放招商年，做到;力向工业集中，加强对工业的组织领导，搞好对工业发展的谋划、协调和指导。按照双向选择的原则，市县领导挂钩服务2—3家本地大型企业或外来投资的大企业，实行全程跟踪服务。对投资超10亿元的项目，成立市级领导牵头的领导小组，提供全方位服务。做到资源向工业汇集，坚持多措并举，实施外向带动、民资推动、盘活促动，加速各类要素向工业汇集，扩大工业总量。以做大做强企业、增加就业岗位为目的，以产权制度改革为重点，在重视引进外来要素参与改制的同时，因企制宜、一厂一策，重点抓好44家市属工业企业的改革，年内完成改制22家以上。推进市直国有商贸、物资、供销企业改革，确保按时完成改制任务。促进银企合作，今年新增贷款在去年的基础上有较大提高。加快城市商业银行组建步伐，上半年正式挂牌。加大对中小企业的支持力度，做大市担保中心规模，担保规模达到3亿元以上。做到政策向工业倾斜，把培育扶持骨干企业、壮大优势产业和建设工业园区放在突出位置，设立新上项目和规模企业发展奖励基金，对投资总额、销售收入、入库税收达到规定额度的，给予奖励。对列入省重点的项目和高新技术项目，在土地供应等方面给予优先支持。通过实施工业突破，到“十一五”末，第一平台的县市当年人均城镇固定资产投入超万元，有年销售收入50亿元以上的骨干企业或100亿元的优势产业，人均规模工业增加值1万元左右，财政收入达到5—15亿元；第二平台的县市当年人均城镇固定资产投入1万元左右，有年销售收入10亿元以上的骨干企业或50亿元的优势产业，人均规模工业增加值8000元左右，财政收入达到2—5亿元；第三平台的县当年人均城镇固定资产投入8000元左右，有年销售收入5亿元以上的骨干企业或30亿元的优势产业，人均规模工业增加值5000元左右，财政收入达到1—2亿元。

　　（二）突出开放招商，扎实推进项目建设。把项目建设作为带动经济社会发展全局的龙头来抓，在继续实施骨干企业再造工程的同时，以开放招商为主要途径，靠招商引资实现大投入，以大投入带动大发展。首先，狠抓项目建设。把项目建设与优化经济结构、转变增长方式、加快城市化进程、发展社会事业结合起来，继续开展 “项目建设年”活动，加大项目建设推进力度，今年初步安排重点建设项目200个，总投资800亿元，年度计划投资230亿元，其中邢钢、邢机、金能、德龙、旭阳五大企业集团的技改投入分别超过10亿元，建滔、龙海集团投入分别超过5亿元；支持金能集团加快建设北掌、邢北煤矿和玻纤二期工程。搞好项目谋划和前期工作，突出抓好正在谋划的建滔煤化工、美国卡博特40万吨碳黑加工、大成集团玉米化工、沙河煤电一体化等30个投资10亿元以上重大项目的前期工作。其次，全力推进招商引资。大力开展全民招商活动，加大考核和奖惩力度，增加压力、激发动力。树立科学的招商理念，由过去招商引资逐步向“招商选资”转变，注重引进就业容量大、对地方财政贡献度高、产业链条长、带动能力强的项目。改进招商方式，立足本地产业、企业和产品优势，选择国内产业集中度高、企业规模大、科技含量高、与本地产业相近的地方，开展登门招商、定向招商、对口招商等，搞好有效对接，主动承接转移。充分发挥企业的主体作用，鼓励企业围绕品牌、技术、产品、市场等，选择战略合作伙伴，通过优势互补，吸引外资投入。支持邢钢、晶龙在境外上市。围绕招商引资搞好服务，重点抓好实际利用外资 1000万美元以上项目，提高履约率和资金到位率。大力发展外向型经济，促进外贸增长方式转变。

　　（三）加快工业园区建设步伐，促进产业集聚。园区建设是发达地区承接产业转移、加快经济发展的成功经验，符合科学发展观的要求。一是加强工业园区建设。把园区建设作为整合基础设施，促进能源、资源、信息、技术共享，有利于生产要素节约，有利于污染综合治理，有利于提高产业集中度的新型工业化发展之路，每个县市都要规划建设有一定规模的工业园区，引导产业、企业、项目进入园区，形成产品关联度高、侧重专业分工、配套协作性强的块状经济。坚持园区规划与城镇规划相衔接，注重与城镇的融合与功能补充，实现协同发展。坚持新办工业项目尽可能集中进园，把好项目入门关、日常监督关与出口关。坚持环保前置审批，严格控制污染物排放，形成统一管理、集中治污、降低成本的集约发展与循环发展格局。二是加快百里工业经济带发展步伐。今天的投资结构就是明天的经济结构，因此，坚持高起点、高标准谋划，为建设产业结构优、增长质量高、生态环境好的经济隆起带奠定基础，年内重点抓好16个工业园区、6个物流园区、13个生态园区的规划，制定招商引资、环境保护等方面的措施，推进百里工业经济带发展。三是抓好产业集聚。结合园区建设，年内突出抓好7个省重点扶持的产业集群，逐个产业制定发展规划和扶持政策，加强引导，促进产业集聚，提高区域经济综合竞争力。

　　（四）以存量调强、增量调优为原则，加快经济结构调整步伐。一是构筑战略支撑产业。围绕发挥资源、产业优势，重点培育4个增长极：钢制品产业抓好15个标准件、紧固件项目，总投资超10亿元，鼓励邢钢建设紧固件企业孵化园，今年新引进年加工能力万吨以上企业10家，全年线材加工能力达到20万吨，明年达到 50万吨；围绕德龙集团的中宽带板材，发展冷轧薄板、弯头管件等板材深加工产品。煤化工产业抓好建滔焦化二期开工和160万吨甲醇、50万吨烯烃项目谋划，争取早日开工建设，支持旭阳建设煤焦油深加工项目，打造邢台煤化工基地。硅材料产业近年发展迅速，已在国际国内产生了重大影响，继续抓好系列产品开发，延长产业链条，建设世界最大的宁晋太阳能单晶硅生产基地。装备制造业重点支持中钢邢机投资11亿元的大型技改扩能项目，促进邢机装备制造水平达到世界一流。二是促进传统产业优化升级。依托羊绒、玻璃、自行车零部件、轴承、板材、食品加工等传统特色产业，加大投入和对外合作力度，引进和培育骨干龙头企业，带动产业升级。实施品牌战略，开发一批高档次、高质量、高附加值的新产品，用先进适用技术改造提升一批传统产品，促进产品结构升级。做大做强优势骨干企业，不断增强创新能力、发展能力和核心竞争能力，今年优势骨干企业的产值、利税占全部入统工业企业的比重分别提高3—5个百分点。三是培育县域主导产业。从发展阶段、基础条件出发，立;发挥现有优势，打造区域特色，每个县市重点培育1—2个主导产业，实行分类指导、梯次推进。对经济基础弱、主导产业不明显的地方，解决产业“从无到有”的问题。对发展有一定基础、主导产业初步形成的地方，解决产业“从有到好”的问题。对发展较快、具有一定实力、主导产业支撑作用明显的地方，解决产业“从好到强”的问题。四是大力发展服务业。把加快发展服务业与推进工业化、城市化结合起来，以发展生产型服务业、现代物流和大型综合专业市场为重点，推广恒祥、东盛英华物流龙头企业的经验，重点抓好医药、粮食、烟草等物流园区建设；抓好大型企业物流职能的剥离，加快主辅分离；抓好市场建设，积极推进“万村千乡”市场工程。突出发展旅游业，抓好“山水太行、古都邢台”品牌的论证规划、资源的挖掘整合和开发宣传；进一步打造“中国七夕文化之乡”和“郭守敬故里”城市名片，提高我市的知名度和美誉度，推动旅游产业的扩张，带动百里太行生态旅游的开发建设。

　　（五）以增加农民收入为核心，推进社会主义新农村建设。继续坚持“多予、少取、放活”的方针，积极推进现代农业建设，强化农村公共服务，促进农民增收、农业增效。一是培育农业产业化龙头。重点抓好南宫千喜鹤、沙河凯特饲料、巨鹿燕南、宁晋三鹿等投资5000万元以上的农业产业化项目建设，带动产业发展，到年底，全市年销售收入5000万元以上的龙头企业比上年增加5家，农业产业化经营率达到59%。建立完善农业合作组织和行业协会，提高农民组织化程度。二是调整农业结构。在确保粮食生产安全的同时，进一步做大做强畜牧、蔬菜、林果三大主导产业，到年底三大主导产业占大农业的比重达到58%，其中畜牧业比重达到42%；培育扶持林木、食用菌、饲料饲草三个新兴产业。积极推广棉菜、枣棉、林粮间作套种、复合经营等高效种植模式，探索土地适度规模经营，提高产出率和综合效益。加强农业标准、检验检测和产地认证三大体系建设，大力发展优质专用农产品、无公害食品和绿色食品生产，努力提高农产品市场竞争力。三是加强农村基础设施建设。抓好南水北调和大沙河倒虹吸工程；治理山区水土流失面积100平方公里；加快临城水库除险加固和滏阳河中游治理四期工程；发展节水灌溉面积4万亩；抓好防汛、防疫、防火、防虫、防雹工作，增强抵御自然灾害能力。四是加快农村劳动力转移。实施“阳光工程”，促进劳务输出向深度和广度发展，全年培训农村劳动力8万人，实现跨省转移劳动力42万人，异地就业14万人。五是深入开展文明生态村创建活动。把创建文明生态村与建设新农村结合起来，坚持实事求是，注重实效，因地制宜，分类指导，逐步推进，通过转变一批、提升一批、改善一批、迁建一批，促进新农村建设。当前，从群众最急需解决的问题入手，重点抓好农村道路、饮水、村容整治等工作，力争30%的村建成文明生态村。

　　（六）以产业为支撑，加速推进城市化进程。以“完善城市功能，打造宜居城市”为目标，突出重点，打造亮点，完善功能，提升品位，加快城市化进程。一是强化产业支撑。把城镇建设与经济发展和生产力布局相结合，引导企业产业向园区集中、园区向城镇集中、人口向城镇集中。中心城市围绕壮大市本级经济实力，重点支持邢钢、邢机、金能、兴泰、方圆、蓝鸟、晶牛、辰光等骨干企业加快发展、做大做强；抓好市开发区标准件、紧固件及相关产业发展；加大扶持力度，发挥市区原有工业企业的设备、技术、品牌、人才等优势，促其焕发生机；围绕机械制造、发展高新技术、旅游配套设施等，谋划建设一批新项目，形成新的经济增长点。同时，组织指导各县市围绕产业规模化、集约化发展，加大园区谋划和建设力度，引导各类项目、企业向县城和重点镇集中，发挥集聚效应。二是抓好规划编制。加快城镇总体规划修编工作，今年全部完成市、县城镇总体规划的修编任务，中心城区115平方公里的城市总体规划，争取早日获得省政府批复。同时，及早完成土地利用总体规划修编。强化规划的龙头作用、综合调控作用和活跃全局的作用，增强规划执行的“刚性”，禁止违法违章建设。三是提高中心城市承载能力。以七里河新区建设为重点，拓宽城市发展空间，打造城市新形象。当前要加快建设规划的编制和实施，全年投资10亿元以上，重点搞好河道治理，建成滨河的南二环，完成南北岸滨河路路基76万平方米，建设首期2.2公里的样板段，新华路大桥“五一”前建成通车，尽快形成环境优美、交通便捷、基础设施完善的城市新区。围绕完善城市功能，重点抓好北二环、东三环等城市主干线的改造拓宽；下大力抓好中兴大街的拆违美化，形成市区示范路；抓好建筑、生活、医疗垃圾综合处理和便民市场等工程建设；加快供热供气工程建设，新增集中供热面积200万平方米。围绕提升城市品味，注重在市区中心黄金地段建设绿地、游园、广场，把人大原址改建成人民广场；开工建设市图书馆，谋划筹建市博物馆；继续建设改造达活泉公园、森林公园等；以种植乔木为重点，做好城区绿化工作，市区新增绿地面积200 万平方米，绿化覆盖率达到37%，人均公共绿地面积达到8.9平方米。围绕创建省级环保模范城，进一步改善市区环境质量，以“三路一街”为重点，加大对违章建筑的拆迁力度，拆除集中供热区域内燃煤锅炉100台。围绕推进城市管理体制机制创新，实行精细化管理，逐步解决城市“脏、乱、差”的问题，提高城市管理的质量和水平。四是抓好小城镇建设。围绕完善功能，谋划建设一批品位高、特色明显的公园、广场、商贸小区等工程，提高要素吸纳能力，增强辐射带动力。今年全市城市化率达到37.5%。

　　（七）加强基础设施建设，加快构筑西联东出交通枢纽。抓住邯黄铁路已列入国家“十一五”地方铁路建设规划并进入实施阶段的机遇，在铁路沿线谋划建设车站、货场、专用线等设施，为打造“西联东出”物流枢纽奠定基础；开展“交通会战年”活动，进一步抓好邢临、青银高速公路连接线建设，积极做好大广、邢汾高速公路前期工作，力争早日开工建设；认真谋划邢港高速公路，带动环城高速路建设；抓好国省干线公路升级改造，其中107、106线建设标准为双向四车道一级公路；加快“百里工业带、百里旅游带”区域内公路建设，完成393公里等级公路改造；抓好农村公路建设，确保实现村村通油路目标。电力基础设施，完成邢台北 500千伏输变电和守敬、临城220千伏输变电工程等30个项目，为全市经济发展提供基础保证。

　　（八）走新型工业化道路，推进体制机制和科技创新。一是围绕重点行业和优势骨干企业，加强信息技术的开发和应用，加快信息网络建设，推进信息化进程，支持县域特色产业信息化建设，以信息化带动工业化。二是充分发挥企业技术创新的主体作用，重点建设“技术创新、成果转化和信息服务”三大平台，加快推进科技进步和科技成果向市场转化，全年完成企业技改投入112亿元，转化科技成果30项。大力开展科技招商活动，围绕引进、消化和推广，推动企业与科研单位、高等院校开展多种形式的合作，积极引进技术、引进人才。三是加强财税工作，深化财政体制改革，进一步优化财政收入结构，不断提高财政收入占GDP的比重、一般预算收入占全部财政收入的比重、税收占财政收入的比重。同时，以转变政府职能为重点，加快行政管理体制改革，严格控制机构编制增长。不断深化投资体制改革，规范政府投资范围和行为。积极推进事业单位、农村综合改革、商贸流通领域等各项改革。

　　四、加强和谐邢台建设

　　按照关注民生、发扬民主、保障民安、塑造民魂、促进民和的总要求，大力实施“民心工程”，切实解决好群众切身利益问题，为公众提供更多更好的社会公共服务，努力形成人与社会、人与自然和谐相处的局面。

　　（一）推进资源节约型、环境友好型社会建设。一是狠抓节能降耗。重点抓好6家国家监管企业和10家省监管企业节能降耗指标的监控。加快“十大节能工程”建设，抓好钢铁、建材、造纸等高耗能行业和企业技术改造，推广节能先进技术。大力发展循环经济，按照减量化、再利用、资源化的原则，抓好循环经济试点，提高资源利用率。二是加强环境治理和生态建设。把水、大气、土壤作为污染防治的重点，严格执行强制淘汰和限期治理制度，依法关停、搬迁、改造一批重点污染企业，特别要加强对市区大气影响严重企业的环境监管，督促和协助企业限期完成废气治理任务，确保减排目标的实现。严把新上项目环境准入关，控制新污染产生。年内实现COD削减4%以上，二氧化硫削减5%以上，市区二级和好于二级天数达到290天以上，争取超过300天。大力实施一批生态建设项目，注重山区生态保护，合理开发利用资源，全面推进生态市建设。三是节约集约利用土地。加强耕地特别是基本农田保护，禁止违法违规用地、滥占耕地。坚持布局集中、产业集聚、用地集约的原则，按照投资强度和容积率标准审核项目用地，提高土地利用效率。

　　（二）加强就业和社会保障工作。千方百计扩大就业再就业，落实扶持自主创业的政策措施，完善就业服务体系，加强劳动保护，发展和谐的劳动关系，全年城镇新增就业4万人，登记失业率控制在4.5%以内。进一步健全社会保险制度，扩大覆盖面，重点抓好非公有制经济组织人员、城镇灵活就业人员和农民工的参保工作，全年企业养老、医疗、失业、工伤保险新增参保8万人。完善社会救助体系，全面落实新的五保供养条例，城镇低保做到动态管理下的应保尽保，符合条件的农村贫困居民全部纳入低保范围，保障面不低于农业人口的1.3%。完善城乡医疗救助制度，探索多种救助方式相结合的医疗救助模式。及时应对突发性自然灾害，保证灾民基本生活。加大扶贫开发力度，扎实推进扶贫开发工程，深化“四帮一”工作。大力实施农村饮水安全工程，年内解决9万人的饮水安全问题。

　　（三）加快发展各项社会事业。放宽政策限制，鼓励和引导各类经济成分投资参与社会事业建设。一是优先发展教育事业。确保“两免一补”、公用经费、教师工资和危房改造资金按时足额到位。全部免收农村义务教育阶段学杂费，对城乡贫困家庭学生免费提供课本和补助寄宿生活费。加快农村中小学布局调整，改造中小学 80所，新建校舍12万平方米，消除危房3万平方米。大力发展职业教育，与职工就业、农村劳动力转移、发展主导产业相结合，推行工学衔接和校企合作，满足不同人员的学习需求，增强劳动者就业技能，全年转移培训劳动力12万人，培训农村实用技术60万人次，开工建设邢台技师学院。继续抓好高等教育，提高发展水平。二是全面发展文化体育事业。加强社会主义精神文明建设，深入开展爱国主义、社会主义和“八荣八耻”教育活动，提高全民思想道德素质。继续推进文化下乡和“彩色周末”文化活动，丰富群众文化生活；搞好文化保护和历史文化研究，适时启动国家历史文化名城申报工作；加强文化设施建设，重点抓好县级文化馆、图书馆和乡镇文化站的建设。推进全民健身运动，提高竞技体育水平。三是加快发展卫生事业。建设覆盖城乡的公共卫生和基本医疗服务体系，完成3个县级医疗机构和35个乡镇卫生院建设。在抓好宁晋、邢台县试点的基础上，今年全部实行新型农村合作医疗制度。加大传染病预防控制力度，防止重大疫病发生。继续抓好人口和计划生育工作，计生率达到89%以上。同时，抓好统计、档案、气象、人防等工作。

　　（四）全力维护社会稳定。正确处理人民内部矛盾，加强和改进信访工作，加大矛盾纠纷排查调解力度，积极预防和妥善处置群体性事件。发挥民族宗教在促进社会和谐稳定上的作用。深入开展“平安邢台”创建活动，加强社会治安综合治理，建立和完善多方位、立体化、无缝隙的治安防控体系，依法打击各种刑事犯罪，今年内使市区社会治安状况实现好转。下大力整顿规范市场经济秩序，让群众吃上“放心食品”、用上“放心药品”。高度重视安全生产工作，继续深化安全生产集中整治，扎实推进标准化建设，强化监督和管理，层层落实安全生产责任制，遏止重特大事故发生。

　　五、推进政府自身建设

　　面对新形势、新任务，我们要牢固树立科学发展观和正确政绩观，切实加强行政能力建设，讲团结、重科学、尚实干，努力提高驾驭市场经济的能力和水平。

　　（一）加强学习，提高决策水平。各级公务员特别是领导干部要把加强学习作为履行职责的一项基本要求，作为做好工作的一项基本方法，下功夫学习社会主义市场经济基本理论和专业知识，特别是产业经济、产业政策、经济管理、经济调节、市场监管、投资融资、外资外贸等方面的知识，不断深化对市场羌媚谠诠媛伞⒃诵刑氐愕娜鲜叮Щ岚淳霉媛砂焓拢銮吭擞谜摺⒎杉熬玫仁侄喂芾砭煤蜕缁崾挛竦哪芰ΑＨ险嫜芯抗液旯鄣骺氐纫幌盗姓撸钒盐辗⒄沟姆较蚝椭氐悖鹬乜蒲А⒆鹬毓媛桑銮烤霾叩那罢靶浴⒖蒲院驼攵孕裕跎俸捅苊馐螅僮吆筒蛔咄渎罚龅皆诓箍蔚耐笔迪指铣Ｍ晟凭霾咦裳疲⒔∪卮笪侍饧寰霾摺⒆易裳⑸缁峁竞凸さ戎贫龋丫霾咧鸩侥扇牍娣痘⒖蒲Щ斓馈?/P>

　　（二）转变职能，优化发展环境。发展环境的差距在政府部门，优化发展环境，关键在政府部门，任务主要在政府部门。要以提高行政效能为重点，积极推进行政创新，继续抓好市、县行政服务中心完善提高，进一步规范办事程序，实行公开承诺、限时办结等制度，清理行政审批事项，加快行政审批流程，相对集中使用审批权，缩短审批周期，严禁出现推诿扯皮、态度蛮横、效率低下等现象。加强对执法部门的管理，正确行使规范市场和社会秩序的行政手段，严肃查处乱收费、乱罚款等行为。加大对企业周边环境的综合整治力度，严肃查处破坏投资环境、侵害投资者利益的人和事，确保投资者的合法权益不受侵犯，在全社会形成“亲商、护商、帮商”的良好氛围。

　　（三）改进作风，狠抓工作落实。增强加快发展的使命感、责任感、紧迫感，以奋发有为、事争一流的精神状态推进工作开展，努力在各个领域创优争先。坚持重实际、说实话、办实事、求实效，不图虚名，不务虚功。大力推进政府诚信建设，确保政府承诺落到实处。密切联系群众，精简会议，压缩文件，用更多的精力和时间，深入基层，调查研究。推广电子政务，提高行政效率。加强以抓落实为核心的作风建设，强化责任，加大督查和考核力度，实行行政问责制，在全市上下形成务实创新、苦干实干、奋力争先的良好风气。

　　（四）依法行政，自觉接受监督。加强法治政府建设，创新管理方式，整合行政资源，降低行政成本。加强民主法制建设，深化政务公开、村务公开、公用事业单位办事公开制度，扩大基层民主。深入贯彻《全面推进依法行政实施纲要》，认真执行《行政许可法》，按照法定权限和程序履行职权，努力做到依法行政、程序正当、诚实守信、权责统一。全面落实《公务员法》，大力提升政府公务员的整体素质和水平。加强执法队伍建设，落实行政执法责任制、追究制和评议考核制，加快建立权责明确、行为规范、监督有效、保障有力的执法监督体系。自觉接受同级人民代表大会及其常委会的法律监督和工作监督，接受人民政协的民主监督，接受新闻舆论和社会公众监督，诚恳听取民主党派、工商联、无党派人士和各人民团体的意见，形成推动经济社会发展的强大合力。

　　（五）清正廉洁，树立良好形象。始终牢记“两个务必”，大力发扬谦虚谨慎、艰苦奋斗的精神，坚决制止铺张浪费。努力克服形式主义和官僚主义，切实纠正部门和行业不正之风，认真解决各种损害群众利益的问题。充分发挥监察、审计等监督机关的作用，强化对行政机关履行职责的监督检查。严格执行党风廉政建设责任制，抓好行政权力公开透明运行、治理商业贿赂等工作，深入开展反腐败斗争，加大查办案件力度，切实解决群众反映强烈的突出问题，树立清正廉洁的良好形象。

　　各位代表！站在新起点，抢抓新机遇，打造新优势，实现新跨越，是时代的呼唤，人民的重托，是历史赋予我们光荣而神圣的使命。让我们在省委、省政府和市委的正确领导下，紧紧依靠全市人民，同心同德，顽强拼搏，乘势而上，为加快实现“富民、强县、兴市”的目标而努力奋斗！
